# Advancing Medical Research Through Artificial Intelligence: Progressive and Transformative Strategies: A Literature Review

**DOI:** 10.1002/hsr2.70200

**Published:** 2025-02-19

**Authors:** Ahmad R. Al‐Qudimat, Zainab E. Fares, Mai Elaarag, Maha Osman, Raed M. Al‐Zoubi, Omar M. Aboumarzouk

**Affiliations:** ^1^ Department of Surgery, Hamad Medical Corporation Surgical Research Section Doha Qatar; ^2^ Department of Public Health, College of Health Sciences, QU‐Health Qatar University Doha Qatar; ^3^ Department of Biomedical Sciences, College of Health Sciences, QU‐Health Qatar University Doha Qatar; ^4^ Department of Chemistry, College of Science Jordan University of Science and Technology Irbid Jordan; ^5^ School of Medicine, Dentistry and Nursing The University of Glasgow Glasgow UK

**Keywords:** AI technology, artificial intelligence, deep learning, healthcare workers, machine learning, medical research

## Abstract

**Background and Aims:**

Artificial intelligence (AI) has become integral to medical research, impacting various aspects such as data analysis, writing assistance, and publishing. This paper explores the multifaceted influence of AI on the process of writing medical research papers, encompassing data analysis, ethical considerations, writing assistance, and publishing efficiency.

**Methods:**

The review was conducted following the PRISMA guidelines; a comprehensive search was performed in Scopus, PubMed, EMBASE, and MEDLINE databases for research publications on artificial intelligence in medical research published up to October 2023.

**Results:**

AI facilitates the writing process by generating drafts, offering grammar and style suggestions, and enhancing manuscript quality through advanced models like ChatGPT. Ethical concerns regarding content ownership and potential biases in AI‐generated content underscore the need for collaborative efforts among researchers, publishers, and AI creators to establish ethical standards. Moreover, AI significantly influences data analysis in healthcare, optimizing outcomes and patient care, particularly in fields such as obstetrics and gynecology and pharmaceutical research. The application of AI in publishing, ranging from peer review to manuscript quality control and journal matching, underscores its potential to streamline and enhance the entire research and publication process. Overall, while AI presents substantial benefits, ongoing research, and ethical guidelines are essential for its responsible integration into the evolving landscape of medical research and publishing.

**Conclusion:**

The integration of AI in medical research has revolutionized efficiency and innovation, impacting data analysis, writing assistance, publishing, and others. While AI tools offer significant benefits, ethical considerations such as biases and content ownership must be addressed. Ongoing research and collaborative efforts are crucial to ensure responsible and transparent AI implementation in the dynamic landscape of medical research and publishing.

**Abbreviations**
AI: artificial intelligence
ChatGPT: chat generative pre‐trained transformer
DL: deep learning
LLM: learning language model
ML: machine learning
NLP: natural language processing

## 1 Introduction

Artificial intelligence (AI) is defined as machine intelligence, as opposed to human or other life forms of intelligence. It is a comprehensive term for computing innovations that mimic human intelligence's supporting systems, including thinking, deep learning, engagement, adaptation, and sensory perception [1]. Research in the field of AI is extensive and diverse, covering areas such as search algorithms, knowledge graphs, natural language processing, expert systems, evolution algorithms, machine learning (ML), deep learning (DL) [2]. With the development of graphical processing units and machine learning technology in the twenty‐first century, the application of AI technology for big data analysis has become widespread in multiple sectors, including medical research. Large language models (LLMs) and AI have become prevalent in medical research, providing new opportunities for idea generation, writing manuscripts, data analysis, and research summarization. Moreover, to minimize technical jargon, it is recommended that clinicians understand additional information about AI techniques applied in medicine. While AI has many different definitions, from decision‐making systems to robots that mimic cognitive processes, ML and DL are two particular types of AI that automate intellectual work [3].

Medical research stands at the intersection of two dynamic domains: the expanding capabilities of AI and the rapidly developing scientific knowledge. The use of AI tools in the production of medical research papers signifies the remarkable convergence of technology and scientific investigation. Beyond accelerating research projects, the various applications of AI, including writing assistance, data analysis, ethical considerations, and simplified publishing, have profoundly changed the research ecosystem.

The complexity and voluminous data inherent in medical research necessitate rigorous methodologies and a meticulous approach to writing. AI‐driven algorithms have become indispensable for analyzing vast and intricate datasets that serve as the foundation for contemporary medical research [4]. Researchers now use machine learning and deep learning models to extract insights from massive amounts of data with remarkable accuracy and speed [5].

Furthermore, AI has significantly influenced the writing process of medical research papers. AI technologies are increasingly being utilized to assist researchers in writing, providing valuable information and support throughout the entire process, thereby enhancing both efficiency and quality. Natural language processing (NLP) algorithms, such as Generative Pre‐Trained Transformer GPT‐3, aid researchers in drafting well‐structured, coherent, and informative manuscripts [6]. Additionally, NLP and GPT models produce easily readable, stylistic, and reproducible medical writing across various specialties, resembling human‐created content [7]. AI‐powered tools facilitate the creation of summaries and abstracts for diverse audiences, from fellow researchers to the general public [8].

Addressing ethical considerations is paramount in medical research, and AI is emerging as a key tool for advancing ethical behavior. Discussion of the ethical difficulties associated with applying machine learning in healthcare is essential for realizing the advantages [9]. Some ethical issues, such as the potential reflection of human biases in algorithms, are evident and need to be addressed [10]. Others, like concerns about data privacy, may pose less evident dangers but raise more general ethical questions [11].

Finally, AI‐driven solutions have expedited and improved access to the publication of medical research. Peer review and manuscript submission procedures are becoming more automated, which lowers administrative overhead and speeds up the dissemination of important research topics. Automated recommendation systems that use AI pair articles with relevant journals and reviewers to increase the likelihood that they will be accepted for publication [12]. In this paper, we delve into the multifaceted impact of AI on the process of writing a medical research paper through discussing data analysis, ethical considerations, writing assistance, and publishing.

## 2 Methods

### 2.1 Literature Search

#### 2.1.1 Information Source and Search Criteria

A comprehensive search was performed in Scopus, PubMed, EMBASE, and MEDLINE databases for research publications on artificial intelligence in medical research published up to October 2023 using the terms were used: “artificial intelligence,” “machine learning,” “deep learning,” “AI technology,” “medical research,” “healthcare workers,” “diagnosis/prognosis, “cancer detection,” “medical imaging analysis,” “drug discovery/development,” and “precision medicine.”

#### 2.1.2 Eligibility Criteria

All included articles encompassing original articles, letters to the editor, editorials, and case reports in English. Recognizing the current limited availability of evidence, preprints, in‐press papers, and research accepted for publication were also considered.

#### 2.1.3 Study Selection and Data Collection

Two writers (A.R.Q. and R.M.Z.) screened and assessed all relevant article texts to ensure that they met the inclusion criteria. Where there were differences of opinion, the senior author (O.M.A.) was consulted until a consensus was established. We only included the most recent papers to minimize duplication of previously published studies by the same authors/institutions.

#### 2.1.4 Search Findings Flow Chart

**Figure 1:**
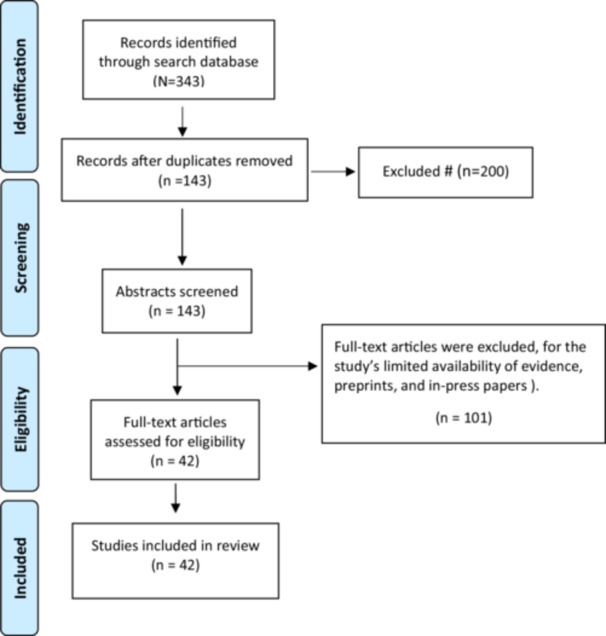
Selection studies flow‐chart.

A cumulative total of 343 studies were identified in the literature review from databases such as PubMed, Scopus, Embase, and Cochrane. Of these, 200 studies were excluded due to duplication, and an additional 101 studies were excluded for the study's limited availability of evidence, preprints, and in‐press papers detailed in Figure 1. Consequently, 42 studies were included in the review. The study screening process is illustrated in Figure 1.

## 3 Results

### 3.1 Using AI in Writing/Rewriting Manuscript

AI has emerged as a powerful tool that is revolutionizing various aspects of medical research, including the writing of research papers. AI technologies are increasingly being utilized to assist researchers in writing medical research papers, offering valuable support throughout the entire process [13]. Artificial intelligence cannot come up with original ideas, but it may arrange and develop the researcher's thoughts to produce a first draft. However, given that the automatically generated language is by no means a replacement for the expertize, originality, and critical thinking of human specialists, this appears to be the beginning of a human‐based development of the text [14]. All stages of manuscript production, from creating first outlines to polishing final manuscripts, can be facilitated by these tools [10]. AI can offer input on grammar, syntax, and style, assisting authors in clear and successful communication of their results [15].

Recent technological advancements have led to the development of sophisticated AI models, some examples are Chat Generative Pre‐Trained Transformer (ChatGPT), Paperpal, Genei, Researcher life, Rytr, and Clickup. These are websites that contain a subset of chatbots and NLP technologies [7, 16]. These models can perform a range of language tasks and generate human‐like responses, which offers exciting prospects for academic efficiency [17]. They operate using algorithms designed to comprehend natural language inputs and respond with the appropriate pre‐written or AI‐generated responses [14]. Chatbots can already assist medical researchers and scientists in generating an entire paper's first draft by writing articles, abstracts, summarizing data or information from literature reviews, suggesting a text's structure, citations, and titles [8]. In addition, text can be generated via a wide range of subjects. The development of abstracts represents a promising area for AI in manuscript authoring [18]. Medical research articles require abstracts since they frequently serve as the readers' first introduction to the article [19]. However, it might be difficult to write an abstract that is both clear and simple, but AI can review the paper and produce an abstract that accurately summarizes the study's findings and complies with the target journal's requirements [8]. According to the author's experience, once the manuscript has been finalized, AI tools are extremely effective for the editing process. It does this through formatting and language editing, rewriting a particularly complex sentence in a clearer way, and even summarizing the entire text to create a suitable abstract [14].

In addition, AI tools have been used to help in plagiarism detection by comparing the text's similarity to already‐published sources, assisting in the verification of the manuscript's originality and lack of plagiarism. NLP technology can be used by chatbots to scan academic papers and offer suitable citation suggestions for found sources As it can assist researchers in identifying the sources they ought to be citing and recommend the right formatting for the citations, this tool has the potential to dramatically streamline the citation process and eliminate errors [6]. Additionally, AI tools can assist researchers in finding fresh sources of data and staying current with advancements in respective domains. In this scenario, a researcher would ask a chatbot, “How do I cite a journal article using the APA citation style?” and it would provide the proper citation format and offer illustrations of relevant papers to quote. In the same manner, they can assist editors in completing repetitive or tedious tasks, like correcting grammatical errors [20]. As we move through the era of digital metamorphosis, AI becomes more and more crucial in significantly enhancing the caliber of scientific writing in many ways. Grammarly and Hemingway are examples of writing enhancement programs that improve syntax and style while fixing spelling problems [21]. They also improve the writing style, offering shorter wording for difficult sentences and pointing out repetitions, resulting in interesting and approachable study content that is easier to read and understand [22].

Overall, AI software tools could potentially be able to aid with the writing process of a scientific paper for medical research publications. These tools can support tasks including formatting, citation, summarizing, and language review as well as tasks like helping to identify research questions and conduct a literature review.

### 3.2 Medical Research Ethics Using AI

The creation of ChatGPT and other AI‐driven technology entails enormous power and responsibility. In light of this, ethical considerations must be taken into account. Although the enormous internet data set used by ChatGPT provides a diverse spectrum of viewpoints and opinions, computational linguist Emily Bender has emphasized that size does not always imply diversity [9]. Furthermore, AI‐driven language models have been compared to “stochastic parrots,” repeating what they hear and frequently distorting it [8]. Research articles written using ChatGPT may be considered unoriginal and perhaps problematic from an ethical perspective. Therefore, any ethical evaluation of ChatGPT must consider its capacity for distortion. Human science has relied on verifiable data and a well‐established system of checks and balances for thousands of years to assure correctness and justice in study findings. A threat to the integrity of science is posed by the growing usage of AI‐generated research articles, which may swiftly produce vast numbers of them and may contain biases and flaws that are challenging to spot and fix [10]. As a result, research findings may become even more unequal, and the tenets of science may be threatened.

The ethical issues surrounding the usage of GPT/ChatGPT, such as content ownership and copyright compliance, apply to AI, NLP, and chatbots as a whole. The usage of third‐party content in the generated texts could raise copyright issues, according to [23]. It is crucial to make sure that any quotes, information, or other resources used in the language model adhere to all applicable copyright regulations and are properly cited [24]. Depending on the type of use, it can be necessary to ask the proprietor of the copyright for permission or to rely on a defense like fair use. It can be difficult to determine how much data from the training set will be quoted or otherwise used in the output produced by a program like ChatGPT.

Copyright concerns lead to plagiarism concerns, and AI has a history of plagiarism in journalism. As a result, there has been much debate about the morality of utilizing ChatGPT to produce scientific articles [25]. The paraphrasing of language, procedures, visuals, ideas, and any other intellectual property that belongs to another person is also considered plagiarism [26]. However, citation practices are promoted in scholarly publishing, and ChatGPT‐generated texts are based on existing literature. Therefore, plagiarism may not be present if the original authors are acknowledged in the text [27].

The future of scholarly and medical publications could be significantly impacted by AI and associated technologies. The ethical implications of these technologies must be carefully considered, especially in light of the usage of GPT‐3 by scientists. It is crucial to make sure that ChatGPT and GPT‐3 are utilized ethically and responsibly for scholarly research and publishing even if they represent significant developments in AI, ML, and NLP. There are still many unanswered challenges regarding the morality of employing GPT in research settings and how it affects the output of research. To ensure the ethical, transparent, and accountable use of emerging technologies, researchers, publishers, and the creators of AI‐driven language models must work together to define standards and guidelines. Failure to do so could erode public confidence in science and have serious repercussions for future innovation and research.

Artificially intelligent medical consultations will significantly impact the physician‐patient relationship. The extent of this impact will depend on how the technology is implemented and adopted. If both physicians and patients embrace AI as a supportive tool that enhances the physician‐patient relationship and remains patient‐centric, AI‐driven medical data could help achieve the vision of personalized or patient‐specific medicine. This approach would allow a properly trained “rational artificial agent” to understand the unique characteristics of a patient's circumstances and inform the physician's strategies and procedures accordingly [28, 29]. The most effective engagement between patients, physicians, and their data is often hindered by restricted access. To enhance data accessibility, a platform that facilitates public visualization of federal, state, and hospital data in a patient‐centered manner is required. This aims to better inform the public through increased transparency, empowering both patients and providers [30, 31]. Electronic Health Records (EHRs) offer a comprehensive patient history that ensures best practices for care. The increased availability of EHRs can reduce redundant testing and save money. Moreover, the data can be used to improve medical practices and track patient outcomes. Physicians can utilize structured data from picture‐archiving communication systems (PACS) to more effectively treat their patients. Additionally, the structured light imaging data within PACS provides an opportunity to leverage artificial intelligence technology to more easily identify pathologies [32, 33].

Data‐driven healthcare refers to the practice of using healthcare data to increase efficiency, improve medical outcomes, and reduce costs [34].

### 3.3 Effects of AI on Data Analysis

According to Jiang et al. (2017), stated that in recent years there has been a rapid increase in the use of data analytic methods due to the application of AI in healthcare. The same authors also stated that the road map of artificial intelligence is from clinical data generation to natural language processing data enrichment, to ML data analysis, to clinical decision‐making [35]. Big data is overwhelming due to its volume, diversity, and speed it must be managed in. Therefore, data analytics has been implemented in the healthcare sector which leads to high‐quality care and lower costs. Data analytics in healthcare can improve outcomes by analyzing patient characteristics, cost, and outcomes, identifying cost‐effective treatments, proactively identifying individuals for preventative care, identifying disease profiling, collecting and publishing medical procedures data, detecting fraud, and implementing real‐time claim authorization. This can also create new revenue streams by aggregating patient records and claims data sets for third‐party services [36]. In addition, data analysis is used in research and development which improves the design of clinical trials and patient recruitment with statistical tools and algorithms to better match therapies to specific patients. Public health focuses on analyzing disease patterns, tracking outbreaks, developing targeted vaccines, and turning large amounts of data into actionable information for population benefit, enhancing surveillance and response [17].

Artificial intelligence has recently been applied to medical research including fields like Obstetrics and Gynecology (OB/GYN). This comprises several fields such as gynecological surgery, fetal ultrasound, and assisted reproductive care that use information from textual, imaging, genetic, and proteomic data sources as well as information from prenatal monitoring. According to Dhombres et al. (2022), there has been a rise in publications related to AI in OB/GYN. Nonsymbolic AI is commonly used in method/algorithm development, hypothesis generation, and software development. In this field, machine learning (ML) has taken the lead through artificial neural networks (ANNs) which mainly deal with ultrasound imaging (2D, 3D, and 4D), numerical, and clinical data sets. Building prediction algorithms for things like implementation success, premature birth, fetal weight, postpartum problems, or neonatal outcome was AI's contribution. Meanwhile the symbolic AI approach includes rule‐based and semantic reasoning, knowledge representation, and formal logic. These strategies don't require a lot of data to generate, require human curation and design, and are typically understandable and human readable. It focuses on knowledge‐based methods like Omics data sets [37].

In the case of pharmaceutical research and development, AI is shown to make drug development more efficient by reducing costs and shortening the development time which leads to an increase in the probability of success. Its main focus is on clinical trial designs, conduct, and analysis. AI/ML‐based clinical trials have especially been beneficial during the COVID‐19 pandemic, where there was an increase in reliance on collecting patient data and the majority of the time clinical trials were conducted virtually [38]. Kolluri (2021) believes that clinical trials can benefit greatly from the application of AI/ML, particularly when it comes to data processing, drafting clinical study reports, and data analysis. Data analysis conducted by AI/ML is compared to a traditional interference‐based statistical approach. By analyzing the drug's benefit‐risk profile and identifying patient subgroups who may benefit more from one treatment than others; biometric data analysis from wearable devices is also utilized to better drug development [38]. Clinical trial data analysis refers to data management, statistical programming, and statistical analysis of patient clinical data collected from trial [39]. In Phase 0 trials and during small‐ Medium dimensional data for evidence generation & Translational research both traditional statistical methods and ML methods are being used in 2022. However, in Phase 1–4 trials that are focused on inference only traditional statistical methods are used. Meanwhile, in translational research or drug discovery data analysis, small‐medium dimensional data uses traditional statistical methods and ML while high dimensional data uses both ML and AI. Adding to this, the development of systems with human‐like reasoning to optimize drug development processes is primarily conducted by AI or DL [38].

### 3.4 Use of AI in Publishing

Although AI technology has been around for decades it is only relatively recently that it has gained widespread recognition and popularity for the capability of its use in writing and publishing articles and research [40]. AI already plays some key roles in the marketing, distribution, and data‐handling aspects of the publishing industry. Additionally, technology has and is being developed that will be able to support and perhaps enhance every aspect of the writing and publishing process and automate several of those tasks [41]. This could potentially result in a massive amount of time being saved on the more laborious aspects of the process.

Opportunities for the use of AI in publishing include “Peer review (including the suggestion of appropriate reviewers), quality control of manuscripts, and identifying suitable journals for a submission.” Several programs have been commercially developed to help with these tasks [41].

In terms of the initial quality control of a manuscript, AI can be used by authors before submission to check for plagiarism and statistical errors, with tools such as ithentaicate [42] and StatCheck. Furthermore, there is the ability to perform a multipurpose manuscript evaluation [43] using tools such as the AuthorOne website [44] to assess if the manuscript is written and formatted appropriately for submission and Frontiers AI review assistant which uses automated quality checks to ensure that the manuscript is ready to be sent for peer review and journal ready. Once the manuscript can be considered as ready for publication there are also online AI tools such as Trinka and Elsevier Journal Finder [45, 46] that act as Journal Finders using the article abstract and title with “concept‐matching technology” to ensure the paper is in line with the journal standards and scope, to avoid journal rejection and consequently get published successfully.

AI is a powerful tool in research and publishing, solving many critical problems in modern science. The use of AI in scientific research and publishing is extensive and requires thorough study. AI‐based language correction systems will likely replace traditional ones due to their inherent advantages and ongoing improvements in technology. These developments will enhance the coverage and quality of language technologies across various languages and improve many other areas of science and education. There have also been suggestions that post‐publication peer review can be conducted using AI, however, it is not yet widely accessible due to the shortage of data (postpublication peer reviews for analysis) available which is required for the AI tool to function effectually. While the potential advantages of its use in publishing are evident there are similarly serious concerns that need to be considered such as data privacy, security, quality, bias, and ethics when utilizing AI technology [43, 47, 48].

AI has already begun to be employed by manuscript authors to help streamline and speed up the submission and publication process and will most likely continue to play an increasing role in both the creation of content and the publication and post‐publication process as it continues to evolve and innovate, additional research and guidelines for its implementation and benefit will certainly be essential.

### 3.5 Use of AI in Diagnosis and Prognosis

AI technologies are widely employed in disease diagnosis and prognosis. Over the years, medical imaging techniques have played a crucial role in diagnosing various diseases and monitoring patient responses to therapies. Cardiac applications, in particular, heavily rely on ultrasound, a popular imaging technique that uses reflected sonar energy to generate detailed images of the heart [49, 50, 51]. Busy clinicians face a significant challenge when using these medical imaging technologies: they generate an overwhelming amount of data that surpasses the interpretive capabilities of even seasoned radiologists and physicians. Consequently, there is a pressing need for cutting‐edge technologies capable of efficiently evaluating and analyzing this vast wealth of medical imaging information. Researchers and AI scientists are addressing this challenge by leveraging state‐of‐the‐art machine learning technologies to aid physicians in interpreting extensive medical imaging data [52].

AI has been extensively utilized in developing applications for the direct or indirect identification of individuals suffering from heart failure (HF). For direct identification, these applications analyze biomedical data to determine whether a subject is affected by HF. Indirect identification involves using the same biomedical data to predict the likelihood of a subject developing heart failure in the future. An excellent example of direct identification applications is the innovative remote cardiac monitoring system developed by VitalConnect [53]. Purkaystha et al. reveal that this system employs an array of vital indicators such as respiration rate, instantaneous heart rate, posture, skin temperature, activity level, distance walked, and other pertinent biomedical data. By meticulously analyzing this diverse range of information, it becomes possible to predict when a patient with heart failure is exhibiting symptoms of declining health. Remarkably, the early warning signs of heart failure can manifest several weeks before hospitalization becomes necessary [54]. By harnessing the power of AI, medical professionals can enhance their diagnostic accuracy and prognostic evaluations, ultimately improving the quality of care provided to patients with cardiac conditions.

### 3.6 Use of AI in Cancer Detection

AI is revolutionizing cancer detection, enabling earlier identification of potential cancer growth. Early detection is crucial for successful treatment, and AI offers significant advantages for at‐risk individuals. Machine learning algorithms can identify likely tumor growth more accurately and earlier than human doctors. Advances in convolutional neural networks have enhanced image recognition, allowing machines to spot specific visual patterns. This use of existing technology has had transformative effects on the medical industry. Previously, detecting whether a cell was cancerous or benign required prolonged examination under a microscope, with human error being a potential issue. AI eliminates the possibility of human error, providing unprecedented accuracy and reliability in cancer detection [55, 56].

AI not only helps confirm the presence of cancer but also enables doctors to track tumor progression in ways that were previously impossible. By accurately identifying the characteristics and strength of a tumor, advanced machine learning algorithms can efficiently determine whether a patient is in remission or if the cancer is likely to return. Early detection and AI assistance allow doctors to navigate cancer treatment more effectively, reducing the emotional toll of relapses for many patients.

### 3.7 Use of AI in Medical Imaging Analysis

Medical imaging is a primary technology that many doctors rely on for accurate diagnosis, effective treatment, and comprehensive assessment of various medical conditions to ensure the well‐being and safety of their patients. With its capabilities, doctors can detect diseases like cancer, cardiovascular diseases, and neurological disorders at early stages, enabling early intervention and improving treatment success rates. Additionally, medical imaging guides complex procedures, evaluates the effectiveness of medical services, and monitors treatment progress and response. With an aging population, the healthcare industry focuses on improving patient care and achieving positive outcomes by implementing advanced medical image analysis applications such as breast cancer detection, real‐time MRI analysis, multi‐parametric analysis (including parameters like texture, shape, and intensity), and prostate cancer mortality prediction [57].

A vast amount of unstructured medical image data is generated and utilized daily by hospitals, clinics, and researchers. However, analyzing this data is challenging and time‐consuming due to its size, intricate details, and the complexity of human interpretation. This situation has significantly increased the workload of radiologists, making it difficult to meet the growing demand from patients and healthcare institutions. Therefore, there is an urgent need to leverage artificial intelligence and machine learning to automate and streamline the process, providing valuable support and enhancing the expertize of medical professionals. Today, the field of medical imaging has been transformed by the advent of artificial intelligence. AI has revolutionized medical imaging by enhancing image interpretation, streamlining processes, optimizing workflow efficiency, reducing operational costs, facilitating population health management, and contributing to innovative clinical trial designs. The profound impact of AI in medical imaging is paving the way for more accurate diagnoses, personalized treatments, and improved patient outcomes [58, 59].

### 3.8 Use of AI in Drug Discovery and Development

The potential of AI in drug discovery is immense. It enhances the efficiency and effectiveness of clinical trials and strives to redefine healthcare delivery. Leveraging AI's capabilities, the future of medicine promises personalized and targeted medications, offering patients the best possible outcomes in their fight against various diseases [60, 61].

The AI field is revolutionizing drug discovery techniques, driving the development of groundbreaking pharmaceuticals with enhanced efficiency. AI holds immense potential in several critical areas of drug discovery, including the advancement of structure‐based design methodologies for novel compounds, enabling the creation of unique molecular structures with improved characteristics [62, 63].

Additionally, AI aids in predicting and evaluating the beneficial biological activities of these new molecules by scrutinizing their interactions with biological targets, accelerating the identification of promising drug candidates [64].

### 3.9 Use of AI in Precision Medicine

Precision medicine is an innovative approach that utilizes the unique characteristics of an individual's physiology to accurately predict their future health outcomes. Based on these predictions, it provides tailored recommendations and treatments that can significantly impact an individual's well‐being [65, 66, 67]. The applications of this field are vast and diverse, ranging from precision diagnostics and targeted therapeutics to the development of cutting‐edge medical devices and innovative pharmaceuticals. Precision medicine also optimizes drug administration and offers various options for modifying an individual's diet or restoring their health and vitality [68].

The most profound benefit AI brings to medical research is in precision medicine. Advances in AI enable us to explore the complexities of the human body, creating detailed models that encompass its entire functioning. By simulating various scenarios and conditions, we can visualize numerous potential paths the body could take over time [69, 70]. These predictions provide insights into precise diagnostics, aiding in the identification of specific areas within the body for accurate diagnoses. Additionally, these insights help generate highly targeted therapeutics, tailoring treatments to an individual's unique needs. AI allows us to anticipate and understand the effects of genetic mutations, drugs, aging, and injuries [71, 72]. Through these simulated pathways, AI not only highlights the potential consequences of various factors but also identifies potential adversities in the absence of countermeasures. These comprehensive models offer a holistic understanding of the body's intricate pathways, revealing its vulnerabilities and strengths. This knowledge helps strategize and implement necessary precautions to ensure individuals' well‐being and longevity.

AI enhances our ability to design highly specific pathways, leveraging advancements in life sciences and connected technologies. As our monitoring and modeling capabilities grow, so do the possibilities for refining and perfecting personalized medicine. The integration of AI with these methodologies propels medical research forward, driving innovation and fostering a new era of precision healthcare [53].

## 4 Conclusion

In conclusion, the integration of AI in medical research has brought about unprecedented efficiency and innovation, transforming various aspects of healthcare and the research process. AI's impact spans data analysis, writing assistance, and publishing, significantly enhancing the diagnostic capabilities of clinicians, particularly in cardiology and oncology. By automating the interpretation of medical imaging and streamlining workflows, AI reduces the burden on radiologists and ensures timely and precise diagnosis, thereby improving patient outcomes. In cancer detection, AI's advanced algorithms offer early and accurate identification of tumors, facilitating timely interventions and reducing the risk of human error. Moreover, AI‐driven innovations in drug discovery and development expedite the creation of novel therapeutics, while precision medicine leverages AI to tailor treatments to individual patients' unique genetic and physiological profiles. Natural language processing models like GPT‐3 revolutionize the writing process, improving manuscript structure and quality, although ethical considerations such as biases and content ownership require careful attention. Beyond writing, AI enhances clinical decision‐making, aids in clinical trials, and transforms publishing processes. While the benefits of AI in medical research and publishing are clear, ongoing research and ethical guidelines are crucial for responsible AI implementation in this ever‐evolving landscape.

## Author Contributions

**Ahmad R. Al‐Qudimat:** conceptualization, investigation, methodology, formal analysis. **Zainab E. Fares:** conceptualization, investigation. **Mai Elaarag:** investigation, conceptualization. **Maha Osman:** conceptualization, investigation. **Raed M. Al‐Zoubi:** conceptualization, investigation. **Omar M. Aboumarzouk:** conceptualization, methodology, investigation.

## Ethics Statement

The authors have nothing to report.

## Conflicts of Interest

All authors declare there is no conflict of interest.

### 1 Transparency Statement

Ahmad Al‐Qudimat affirms that this manuscript is an honest, accurate, and transparent account of the study being reported; that no important aspects of the study have been omitted; and that any discrepancies from the study as planned (and, if relevant, registered) have been explained.

## Data Availability

The authors confirm that the data supporting the findings of this study are available within the article or its supplementary materials.
